# Twenty years of surveillance for Eastern equine encephalitis virus in mosquitoes in New York State from 1993 to 2012

**DOI:** 10.1186/s13071-018-2950-1

**Published:** 2018-06-25

**Authors:** JoAnne Oliver, Gary Lukacik, John Kokas, Scott R. Campbell, Laura D. Kramer, James A. Sherwood, John J. Howard

**Affiliations:** 1 Department of Health, Central New York Regional Office, State of New York, 217 South Salina Street, Syracuse, NY 13202 USA; 2 School of Agriculture and Natural Resources, Morrisville State College, State University of New York, 80 Eaton Street, Morrisville, NY 13408 USA; 3 Division of Epidemiology, Department of Health, Vector Surveillance Unit, Bureau of Communicable Diseases, State of New York, Room 651, Corning Tower, Empire State Plaza, Albany, NY 12237 USA; 4 Vector Surveillance Unit, Louis Calder Center, Fordham University, 53 Whippoorwill Road, Armonk, NY 10504 USA; 5 Arthropod-Borne Disease Laboratory, Suffolk County Department of Health Services, 360 Yaphank Avenue, Suite 2A, Yaphank, NY 11980 USA; 6 Arbovirus Laboratory, Division of Infectious Diseases, Wadsworth Center, Department of Health, State of New York, 5668 State Farm Road, Slingerlands, NY 12159 USA; 7 School of Public Health, University at Albany, State University of New York, One University Place, Rensselaer, NY 12144 USA

**Keywords:** Eastern equine encephalitis virus, *Aedes*, *Anopheles*, *Coquillettidia*, *Culex*, *Culiseta*, *Ochlerotatus*, *Psorophora*, New York State

## Abstract

**Background:**

The year 1971 was the first time in New York State (NYS) that Eastern equine encephalitis virus (EEEV) was identified in mosquitoes, in *Culiseta melanura* and *Culiseta morsitans*. At that time, state and county health departments began surveillance for EEEV in mosquitoes.

**Methods:**

From 1993 to 2012, county health departments continued voluntary participation with the state health department in mosquito and arbovirus surveillance. Adult female mosquitoes were trapped, identified, and pooled. Mosquito pools were tested for EEEV by Vero cell culture each of the twenty years. Beginning in 2000, mosquito extracts and cell culture supernatant were tested by reverse transcriptase-polymerase chain reaction (RT-PCR).

**Results:**

During the years 1993 to 2012, EEEV was identified in: *Culiseta melanura*, *Culiseta morsitans, Coquillettidia perturbans*, *Aedes canadensis* (*Ochlerotatus canadensis*), *Aedes vexans*, *Anopheles punctipennis*, *Anopheles quadrimaculatus*, *Psorophora ferox*, *Culex salinarius*, and *Culex pipiens-restuans* group. EEEV was detected in 427 adult mosquito pools of 107,156 pools tested totaling 3.96 million mosquitoes. Detections of EEEV occurred in three geographical regions of NYS: Sullivan County, Suffolk County, and the contiguous counties of Madison, Oneida, Onondaga and Oswego. Detections of EEEV in mosquitoes occurred every year from 2003 to 2012, inclusive. EEEV was not detected in 1995, and 1998 to 2002, inclusive.

**Conclusions:**

This was the first time in NYS that EEEV was detected in *Cx. salinarius*, *Ps. ferox* and *An. punctipennis*. The detection of EEEV in mosquitoes every year for 10 years was the longest time span since surveillance began in 1971. The calendar date of the earliest annual appearance of EEEV in mosquitoes did not change during surveillance spanning 42 years.

## Background

*Culiseta melanura* (Coquillett) becomes infected by, and transmits, Eastern equine encephalitis virus (EEEV) during blood feeding [1]. This species feeds preferentially on birds and opportunistically on mammals [2, 3] and occasionally on humans [4, 5]. Other genera and species of mosquitoes common in the northeastern USA have been found to carry EEEV and have been thought to play a role in the transmission of EEEV, including *Coquillettidia perturbans* (Walker) [6], *Aedes canadensis* (Theobald) (*Ochlerotatus canadensis*) [7–9], and *Aedes cinereus* Meigen [9, 10].

In 1971, the first reported case of Eastern equine encephalitis (EEE) in a human in New York State (NYS), in Oswego County [11], prompted the respective health departments to begin annual surveillance for EEEV in mosquitoes [7, 11–13]. In 1971, EEEV was identified in *Cs. melanura*, *Culiseta morsitans* (Theobald), and *Culex restuans* Theobald, in Oswego County [11, 12]. In 1973, EEEV was identified in *Culex pipiens* Linnaeus, in Suffolk County [13, 14]. In 1974, EEEV was identified in *Ae. canadensis* in Oswego County [12]. In 1976, EEEV was identified in *Cq. perturbans* in Oswego County [13]. In 1978, EEEV was identified in *Cs. melanura*, in Suffolk County (Howard JJ, Oliver J, Guirgis S, Woodall JP. Eastern equine encephalitis in Suffolk County, Long Island, New York, 1994. *Proceedings of the 82nd Annual Meeting New Jersey Mosquito Control Association* 1995;82:12–16) [14, 15]. In 1990, EEEV was identified in *Aedes vexans* (Meigen) and *Anopheles quadrimaculatus* Say [16].

From 1971 to 1992, there were detections of EEEV in eight mosquito species within five genera, in NYS [7]; in order of frequency, 159 were from *Cs. melanura*, 18 from *Cs. morsitans*, 11 from *Cq. perturbans*, nine from *Ae. canadensis*, two from *Ae. vexans*, and one each from *An. quadrimaculatus*, *Cx. pipiens,* and from *Cx. restuans*.

The purpose of this work is to report on continued surveillance for EEEV in mosquitoes in NYS, from 1993 to 2012.

## Methods

### Recruitment of counties

Counties decided to participate in mosquito and arbovirus surveillance based on local concern about vector-borne disease and budget. The number of counties that conducted mosquito surveillance and submitted mosquito specimens for virus assay ranged from nine to 43 from 1993 to 2012 (Table 1) [17]. There are 57 counties in NYS exclusive of the five counties that comprise New York City (NYC). In NYC, the Department of Health and Mental Hygiene conducted their own surveillance and testing and their results are not included in this present study.

**Table 1:** Number of counties conducting surveillance for mosquitoes and Eastern equine encephalitis virus in New York State from 1993 to 2012

Year	No. of participating counties
1993	9
1994	9
1995	9
1996	9
1997	9
1998	9
1999	9
2000	21
2001	43
2002	39
2003	29
2004	29
2005	27
2006	32
2007	26
2008	23
2009	15
2010	15
2011	14
2012	13

### Trapping of mosquitoes

Trapping of adult mosquitoes began in May of each year and ended by October. Mosquitoes were collected using light traps supplemented with dry ice [18], gravid traps [19], or diurnal resting boxes [20]. Counties selected trap types depending on which viruses and mosquito species they were interested in collecting. Counties focusing on EEEV used diurnal resting boxes, optimal for collecting *Culiseta*, and light traps, optimal for *Aedes* and *Coquillettidia*. Counties focusing on West Nile virus (WNV) used gravid traps, optimal for collecting *Culex*, and light traps [21]. The geographical distribution of EEE or EEEV in the recent past [14, 16] or of WNV [17, 22] was used to determine the locations of surveillance sites in the current study.

### Identification and sorting of mosquitoes

The identification of adult mosquito specimens was aided with microscopy and based on morphologic features and nomenclature from published methods [23–26]. Male mosquitoes were excluded. When possible, identifications were made to species level. Some identifications were made to group level (Table 2). Each county decided which mosquito species to submit for testing. Females were sorted by species, by physiological status (unfed, blood-fed, gravid), collection site, type of trap, and week. Appropriately sorted specimens were then pooled. Based on methodology, the laboratory decided on the number of mosquitoes per pool. During the years 1993 to 1999, the number of mosquito specimens per pool was 10–100; during 2000 to 2008, 10–50; and during 2009 to 2012, 10–60. Each year, the testing laboratory, based on capacity, determined a total number of pools they would be able to accept from each county.

**Table 2:** Mosquitoes collected and tested for Eastern equine encephalitis virus in New York State from 1993 to 2012

Species	
*Aedes* (*Ochlerotatus*) *canadensis* (Theobald)	
*Aedes* (*Aedes*) *cinereus* Meigen	
*Aedes* (*Aedimorphus*) *vexans* (Meigen)	
*Aedes* (*Finlaya*) *japonicus* (Theobald)	
*Aedes* (*Ochlerotatus*) *abserratus-punctor* group^a^	
*Aedes* (*Ochlerotatus*) *abserratus* (Felt & Young)	
*Aedes* (*Ochlerotatus*) *punctor* (Kirby)	
*Aedes* (*Ochlerotatus*) *atropalpus* (Coquillett)	
*Aedes* (*Ochlerotatus*) *cantator* (Coquillett)	
*Aedes* (*Ochlerotatus*) *dorsalis* (Meigen)	
*Aedes* (*Ochlerotatus*) *communis* group^a^	
*Aedes* (*Ochlerotatus*) *abserratus* (Felt & Young)	
*Aedes* (*Ochlerotatus*) *communis* (De Geer)	
*Aedes* (*Ochlerotatus*) *provocans* (Walker)	
*Aedes* (*Ochlerotatus*) *punctor* (Kirby)	
*Aedes* (*Ochlerotatus*) *sticticus* (Meigen)	
*Aedes* (*Ochlerotatus*) *grossbecki* Dyar & Knab	
*Aedes* (*Ochlerotatus*) *implicatus* Vockeroth	
*Aedes* (*Ochlerotatus*) *intrudens* Dyar	
*Aedes* (*Ochlerotatus*) *riparius* Dyar & Knab	
*Aedes* (*Ochlerotatus*) *sollicitans* (Walker)	
*Aedes* (*Ochlerotatus*) *spencerii* (Theobald)	
*Aedes* (*Ochlerotatus*) *stimulans* group^a^	
*Aedes* (*Ochlerotatus*) *excrucians* (Walker)	
*Aedes* (*Ochlerotatus*) *fitchii* (Felt & Young)	
*Aedes* (*Ochlerotatus*) *stimulans* (Walker)	
*Aedes* (*Ochlerotatus*) *taeniorhynchus* (Wiedemann)	
*Aedes* (*Ochlerotatus*) *triseriatus* (Say)	
*Aedes* (*Ochlerotatus*) *trivittatus* (Coquillett)	
*Aedes* (*Stegomyia*) *albopictus* (Skuse)	
*Anopheles* (*Anopheles*) *earlei* Vargas	
*Anopheles* (*Anopheles*) *punctipennis* (Say)	
*Anopheles* (*Anopheles*) *quadrimaculatus* Say	
*Anopheles* (*Anopheles*) *walkeri* Theobald	
*Coquillettidia* (*Coquillettidia*) *perturbans* (Walker)	
*Culex* (*Culex*) *pipiens*-*restuans* group^a^	
*Culex* (*Culex*) *pipiens* Linnaeus	
*Culex* (*Culex*) *restuans* Theobald	
*Culex* (*Culex*) *salinarius* Coquillett	
*Culex* (*Neoculex*) *territans* Walker	
*Culiseta* (*Climacura*) *melanura* (Coquillett)	
*Culiseta* (*Culicella*) *morsitans* (Theobald)	
*Culiseta* (*Culicella*) *minnesotae* Barr	
*Culiseta* (*Culiseta*) *impatiens* (Walker)	
*Culiseta* (*Culiseta*) *inornata* (Williston)	
*Orthopodomyia* (*Orthopodomyia*) *signifera* (Coquillett)	
*Orthopodomyia* (*Orthopodomyia*) *alba* Baker	
*Psorophora* (*Grabhamia*) *columbiae* (Dyar & Knab)	
*Psorophora* (*Janthinosoma*) *ferox* (Humboldt)	
*Psorophora* (*Janthinosoma*) *mathesoni* Belkin & Heinemann	
*Psorophora* (*Psorophora*) *ciliata* (Fabricius)	
*Toxorhynchites* (*Lynchiella*) *rutilus septentrionalis* (Dyar & Knab)	
*ranotaenia* (*Uranotaenia*) *sapphirina* (Osten Sacken)	

^a^Groups may contain the species indented below the group name

### Testing mosquitoes for virus

From 1993 to 1999, pools of mosquitoes were tested for virus by Vero cell culture (African green monkey kidney cells), according to the method of Srihongse et al. [13] as modified by Boromisa & Grayson [27]. From 2000 to 2012, pools of mosquitoes were tested for virus by amplification of viral nucleic acid [22]. Pools were placed in 2-ml polypropylene microcentrifuge tubes containing a zinc-plated steel bead (Daisy Outdoor Products, Rogers, Arkansas, USA), kept on dry ice, and tested at the Arbovirus Laboratory, Wadsworth Center, Department of Health, State of New York, in Albany. Every year, a portion was tested for the presence of arboviruses by screening on Vero cell culture [28]. If cytopathology was observed, cell culture supernatant was tested for EEEV by a specific reverse-transcription polymerase chain reaction (RT-PCR) [29] as previously used in our Arbovirus Laboratory [28, 30]. RT-PCR was also conducted on the original mosquito pool homogenate [28, 30]. In detail, two sets of primers and probes, each targeting a different region of the EEEV RNA template, were used for testing specimens. First, sequences for the set targeting the E2 gene were provided by the Centers for Disease Control and consisted of forward primer 5'-ACA CCG CAC CCT GAT TTT ACA-3', reverse primer 5'-CTT CCA AGT GAC CTG GTC GTC-3', and probe 5'-6FAM-TGC ACC CGG ACC ATC CGA CCT-TAMRA-3' [31]. A second set was developed in the Viral Encephalitis Laboratory of the Wadsworth Center of NYSDOH and targeted the E1 gene (forward primer 5'-ACA CTA AAT TCA CCC TAG TTC GAT-3', reverse primer 5'-GTG TAT AAA ATT ACT TAG GAG CAG CAT TAT G-3', and probe 5'-6FAM-CGA GCT ATG GTG ACG GTG GTG CA-TAMRA-3' [30]. Assays were performed on the ABI Prism 7000 or 7500 sequence detectors using ABI TaqMan one-step RT-PCR master mix (Applied Biosystems, Foster City, California, USA). Standards were prepared from RNA extracted from EEEV stock that had been amplified on Vero cells and titers determined [28]. Thermal cycling consisted of 48 °C for 30 min for RT, 95 °C for 10 min, and 40 cycles of 95 °C for 15 s and 60 °C for one min. A sample was considered positive if the C_T_ value was less than 40 for both primer sets, and the ΔRn value was more than five times the average ΔRn values for the negative controls. Results were expressed in C_T_ values or relative numbers of plaque forming units (PFU) calculated by linear regression from the standard curve. Pools are processed for EEEV within three to four days of receipt. The EEEV assay had a sensitivity of five gene copies per reaction [30]. Pools of *Cx. pipiens* and *Cx. restuans* were not inoculated onto Vero cells unless EEEV was detected by RT-PCR.

## Results

### Mosquito specimens

**Fig. 1:**
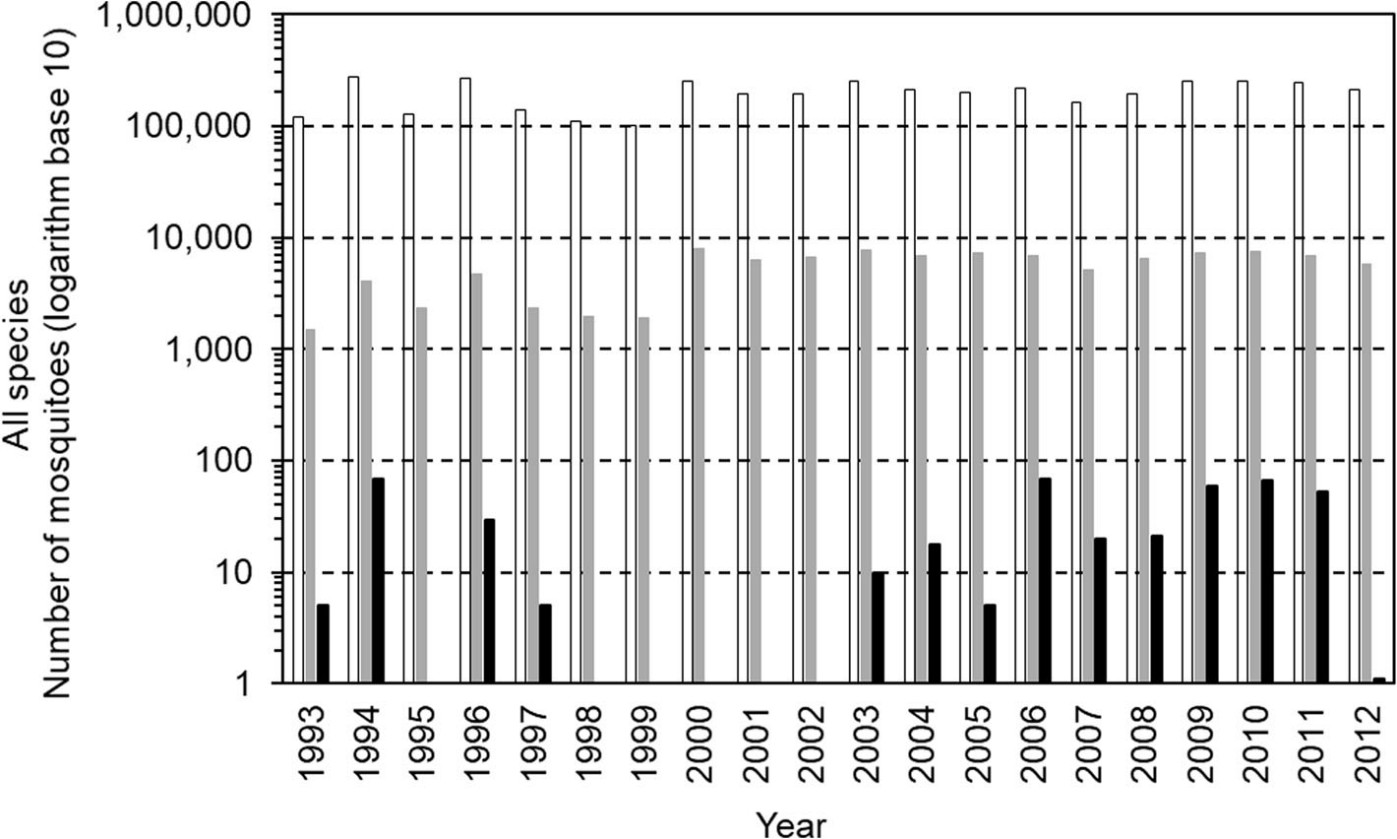
Detections of Eastern equine encephalitis virus in all species of mosquitoes. Bars show the number of mosquitoes submitted (white bars), the number of pools tested (gray bars) and the number of pools positive (black bars), 1993 to 2012, in New York State. From 1993 to 1999, 2000 to 2008, and 2009 to 2012, average pool sizes were 61, 30 and 35, respectively

From 1993 to 2012, statewide, 3.96 million mosquitoes were submitted and assayed (Fig. 1). Of the approximately 70 species of mosquitoes previously known to be present in NYS [23, 24], 49 species were submitted for arbovirus testing (Table 2). There were 11 species of mosquitoes in which EEEV was detected (Table 3). State-wide, *Cs. melanura* accounted for 8.43% (333,871 of 3,960,070) of mosquitoes submitted.

**Table 3:** Number of pools of mosquito species with Eastern equine encephalitis virus from six counties in New York State from 1993 to 2012

	1993	1994	1995	1996	1997	1998	1999	2000	2001	2002	2003	2004	2005	2006	2007	2008	2009	2010	2011	2012	All years (20)
Oswego, Onondaga, Oneida, and Madison counties (4)																					
*Cs. melanura*	0^a^	19^b^	0	2	0	0	0	0	0	0	8	11	4	66	19	16	52	61	46	1	305
*Cq. perturbans*	0	0	0	0	0	0	0	0	0	0	0	1	1	0	0	0	6	4	3	0	15
*Ae. canadensis*	0	0	0	0	0	0	0	0	0	0	1	1	0	0	0	0	1	1	1	0	5
*Ae. vexans*	0	0	0	0	0	0	0	0	0	0	0	1	0	0	0	1	0	0	1	0	3
*Cs. morsitans*	0	0	0	1	0	0	0	0	0	0	0	0	0	0	1	0	0	0	1	0	3
*An. punctipennis*	0	0	n^c^	n	n	n	n	n	n	n	n	n	n	n	n	1	n	n	n	0	1
*Cx. pipiens-restuans*	0	n	n	n	n	n	0	0	0	0	0	0^d^	0	1	n	n	n	n	0	0	1
*Ps. ferox*	n	0	n	n	n	n	n	n	n	n	n	n	n	1	0	0	0	0	n	n	1
*An. quadrimaculatus*	0	0	0	n	n	n	n	n	n	n	n	n	n	n	n	n	n	n	n	0	0
*Cx. salinarius*	n	n	n	n	n	n	n	n	n	n	n	1^d^	n	n	n	n	n	n	n	n	1
Suffolk county (1)																					
*Cs. melanura*	6	43	0	23	5	0	0	0	0	0	1	0	0	0	0	3	0	0	0	0	81
*Cq. perturbans*	0	1	0	0	0	0	0	0	0	0	0	n	0	0	0	0	0	0	0	0	1
*Ae. canadensis*	0	0	0	0	n	0	0	0	0	0	0	n	0	0	0	0	0	0	0	0	0
*Ae. vexans*	0	0	0	0	n	0	0	0	0	0	0	n	0	0	0	0	0	0	0	0	0
*Cs. morsitans*	n	n	0	0	n	n	n	n	n	n	0	n	n	n	n	0	n	n	n	n	0
*An. punctipennis*	0	0	n	0	n	n	n	0	0	0	0	n	0	n	n	n	n	0	0	0	0
*Cx. pipiens-restuans*	0	3^e^	0	3^e^	0	0	0	0	0	0	0	0	0	0	0	0	0	0	0	0	6
*Ps. ferox*	n	0	n	n	n	n	n	0	n	0	0	n	0	n	0	n	n	n	n	0	0
*An. quadrimaculatus*	0	2	n	n	n	n	n	0	0	0	n	n	0	n	n	n	0	0	n	0	2
Sullivan county (1)																					
*Cs. melanura*	-^f^	-	-	-	-	-	-	-	0	n	-	2	0	0	-	-	-	-	-	-	2
*Cq. perturbans*	-	-	-	-	-	-	-	-	0	0	-	0	0	0	-	-	-	-	-	-	0
*Ae. canadensis*	-	-	-	-	-	-	-	-	0	0	-	0	0	0	-	-	-	-	-	-	0
*Ae. vexans*	-	-	-	-	-	-	-	-	0	n	-	0	0	0	-	-	-	-	-	-	0
*Cs. morsitans*	-	-	-	-	-	-	-	-	n	n	-	n	n	n	-	-	-	-	-	-	n
*An. punctipennis*	-	-	-	-	-	-	-	-	0	0	-	n	n	n	-	-	-	-	-	-	0
*Cx. pipiens-restuans*	-	-	-	-	-	-	-	-	0	0	-	0	0	0	-	-	-	-	-	-	0
*Ps. ferox*	-	-	-	-	-	-	-	-	n	n	-	n	n	n	-	-	-	-	-	-	n
*An. quadrimaculatus*	-	-	-	-	-	-	-	-	n	0	-	n	n	0	-	-	-	-	-	-	0
All counties (6)																					
All species	6	68	0	29	5	0	0	0	0	0	10	17	5	68	20	21	59	66	52	1	427

^a^Zero mosquito pools tested positive

^b^Positive integer is number of mosquito pools which tested positive

^c^Mosquito species was not submitted for testing

^d^Mosquito pool was submitted as *Cx. pipiens-restuans* but later determined by molecular analysis to be *Cx. salinarius*

^e^Testing positive in 1994 and 1996 were *Culex pipiens-restuans* and *Culex* not identified to species level

^f^Trapping was not conducted in this county in this year

From 2001 through 2012, in the four counties of Madison, Oneida, Onondaga and Oswego, *Cq. perturbans*, *Cs. melanura* and *Ae. canadensis* accounted for 33%, 17% and 16%, respectively, of all mosquitoes submitted for arbovirus testing. From 2001 through 2012, in Suffolk County, *Cx. pipiens-restuans* group, *Cs. melanura*, *Aedes sollicitans* (Walker) and *Ae. vexans* accounted for 68%, 5%, 5% and 4%, respectively, of all mosquitoes submitted for arbovirus testing.

Whether the number of participating counties was smaller (*n* = 9) or larger (*n* = 43) (Table 1), EEEV was not detected in mosquitoes in the years 1998 to 2002 (Table 4).

**Table 4:** Mosquito specimens pooled and tested for Eastern equine encephalitis virus in New York State, 1993 to 2012

Year	All mosquito species^a^			*Cs. melanura*		
	No. of specimens	No. of pools	No. of EEEV-positive pools (%)	No. of specimens	No. of pools	No. of EEEV-positive pools (%)
1993^b^	119,280	1482^b^	6 (0.34)	67,165	1013^b^	6 (0.5)
1994	275,262	4101	68 (1.66)	43,584	735	62 (8.4)
1995	127,866	2301	0	12,245	449	0
1996	266,685	4659	29 (0.62)	22,766	668	25 (0.04)
1997	140,189	2358	5 (0.21)	19,834	512	5 (0.01)
1998	111,140	1954	0	19,322	385	0
1999	102,755	1911	0	9056	319	0
2000^c^	252,003	7871^c^	0	14,529	395^c^	0
2001	191,981	6360	0	11,349	361	0
2002	194,906	6738	0	6524	222	0
2003	249,554	7714	10 (0.13)	9190	286	9 (3.15)
2004	209,362	6773	17 (0.26)	10,255	302	13 (4.63)
2005	196,371	7178	5 (0.07)	4976	199	4 (2.01)
2006	216,924	6895	68 (0.99)	16,111	482	66 (13.69)
2007	160,886	5155	20 (0.39)	9654	330	19 (5.76)
2008	194,988	6509	21 (0.32)	9785	347	19 (5.48)
2009^d^	250,396	7191^d^	59 (0.82)	15,189	440^d^	52 (11.82)
2010	249,120	7409	66 (0.89)	12,849	417	61 (14.63)
2011	241,447	6767	52 (0.77)	11,725	446	46 (10.31)
2012	208,955	5830	1 (0.02)	7763	298	1 (0.34)
Total	3,960,070	107,156	427	333,871	8606	388

^a^Mosquito species submitted for arboviral testing include *Cs. melanura*

^b^Pool sizes were 10–100 mosquito specimens from 1993 to 1999

^c^Pool sizes were 10–50 mosquito specimens from 2000 to 2008

^d^Pool sizes were 10–60 mosquito specimens from 2009 to 2012

### Mosquito pools

The 3.96 million mosquitoes were assayed in 107,156 pools (Table 4). One to two percent of samples testing positive for EEEV by RT-PCR were not infectious on Vero cell culture. There were 14 of 20 years with EEEV detected in mosquitoes (Table 4). In those 14 years, the percent positive pools ranged between 0.02-1.66% (mean 0.54%) (Table 4). For *Cs. melanura* alone, there were 14 of 20 years with EEEV detected in mosquitoes, ranging between 0.01-14.63% (mean 5.77%) (Table 4). Over the 20 years, the total number of mosquitoes and the total number of pools were within the same order of magnitude (Table 4, Fig. 1).

Detections were made in the following mosquito species: *Cs. melanura*, *Cs. morsitans*, *Cq. perturbans*, *Ae. canadensis*, *Ae. vexans*, *Anopheles punctipennis* (Say), *An. quadrimaculatus*, *Psorophora ferox* (Humboldt), *Culex salinarius* Coquillett and *Cx. pipiens-restuans* group.

**Fig. 2:**
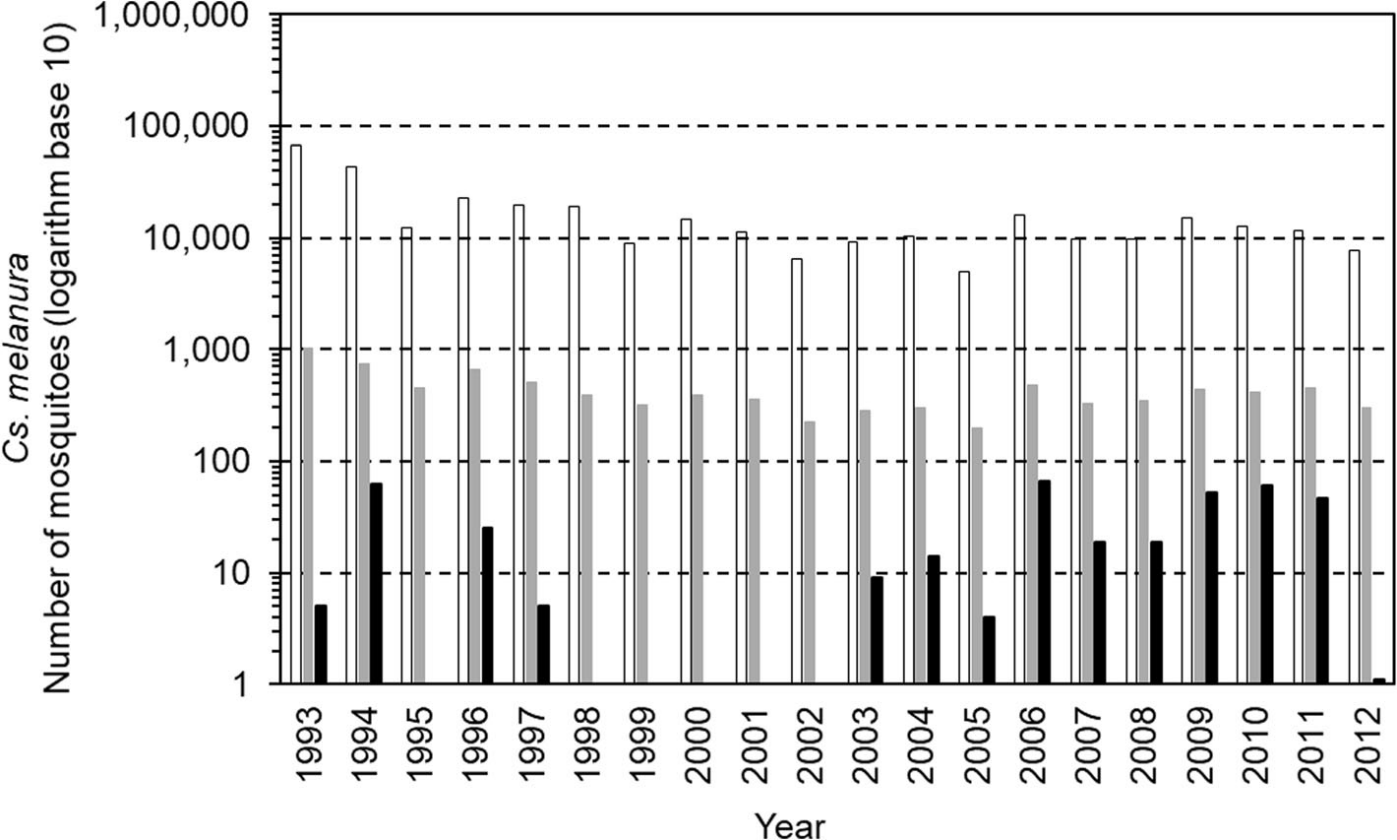
Detections of Eastern equine encephalitis virus in *Culiseta melanura* mosquitoes. Bars show the number of mosquitoes submitted (white bars), the number of pools tested (gray bars) and the number of pools positive (black bars), 1993 to 2012, in New York State. From 1993 to 1999, 2000 to 2008, and 2009 to 2012, average pool sizes were 43, 31 and 29, respectively

*Culiseta melanura* accounted for 91% (388 of 427) of pools in which EEEV was detected (Table 4). The number of *Cs. melanura* pools assayed, and the numbers of pools testing positive for EEEV, each year in NYS from 1993 to 2012, is illustrated in Fig. 2. Species other than *Cs. melanura* accounted for 9% (39 of 427) of pools in which EEEV was detected (Table 4).

From 1993 to 1999, when pool sizes ranged from 10 to 100 specimens, the average pool size was 61 specimens per pool. From 2000 to 2008, when pool sizes ranged from 10 to 50, the average pool size was 30 specimens per pool. From 2009 to 2012, when pool sizes ranged from 10 to 60, the average pool size was 35 specimens per pool. For *Cs. melanura*, the average pool sizes during these periods were 43, 31 and 29, respectively.

### Geographical distribution of mosquitoes with virus

Six counties had mosquitoes that tested positive for EEEV, and these counties were located in three geographical regions of NYS (Table 3).

First, in Madison, Oneida, Onondaga and Oswego Counties, which all have Oneida Lake as a border, EEEV was detected in eight species of mosquitoes and *Cx. pipiens-restuans* group, among 27 species tested from this region. This area yielded 23% (25,071 of 107,156) of all mosquito pools, and 78% (335 of 427) of all EEEV-positive pools. This area also yielded 79% (305 of 388) of all *Cs. melanura* pools that were EEEV-positive, and 77% (30 of 39) of all pools other than *Cs. melanura* that were EEEV-positive.

Secondly, in Suffolk County, EEEV was detected in three mosquito species and one group (Table 3). There were 90 pools in which EEEV was detected, consisting of 81 pools of *Cs. melanura*, six of *Cx. pipiens-restuans* group, two of *An. quadrimaculatus* and one of *Cq. perturbans*. The easternmost location in NYS in which EEEV was detected was the Town of East Hampton, Suffolk County.

Thirdly, in Sullivan County, EEEV was detected in one species of mosquito, *Cs. melanura*, in two pools, in the Town of Thompson (Table 3).

### Timing of mosquitoes with virus

EEEV was detected in mosquitoes in 1993 through 1997, and in 2003 through 2012 (Fig. 1), within at least one of these three regions of NYS. However, EEEV was not detected in all three regions in the same years, during the 20 years of surveillance. EEEV was not detected in mosquitoes in 1995, and 1998 through 2002 (Table 4, Fig. 1). This pattern was the same with *Cs. melanura* alone (Table 4, Fig. 2). EEEV was detected at least once in each of the nine species of mosquitoes and the *Cx. pipiens-restuans* group (Table 3). There was no year during which EEEV was detected simultaneously in all nine species of mosquitoes and the *Cx. pipiens-restuans* group (Table 3).

State-wide, the earliest date in any year that EEEV was detected was June 25 (2007), in Oswego County, and was July 7 (1994), in Suffolk County, and was September 2 (2004), in Sullivan County, all in *Cs. melanura*. Within the 20-year period, detections of EEEV occurred in June (*n* = 2), July (*n* = 81), August (*n* = 234), September (*n* = 108) and October (*n* = 2) (Table 5).

**Table 5:** Years and months of detections of Eastern equine encephalitis virus in mosquitoes during surveillance in New York State from 1993 to 2012

Year	June	July	August	September	October	Total
1993	0	0	4	0	2	6
1994	0	13	35	20	0	68
1995	0	0	0	0	0	0
1996	0	4	17	8	0	29
1997	0	0	3	2	0	5
1998	0	0	0	0	0	0
1999	0	0	0	0	0	0
2000	0	0	0	0	0	0
2001	0	0	0	0	0	0
2002	0	0	0	0	0	0
2003	0	0	2	8	0	10
2004	0	2	7	8	0	17
2005	0	4	1	0	0	5
2006	0	10	44	14	0	68
2007	1	15	2	2	0	20
2008	0	0	10	11	0	21
2009	0	12	33	14	0	59
2010	1	14	41	10	0	66
2011	0	7	34	11	0	52
2012	0	0	1	0	0	1

It was in 1996 and 2003 that EEEV in mosquitoes was detected in the most easterly area of NYS, in the Town of East Hampton, Suffolk County on Long Island. It was in 2004 that EEEV in mosquitoes was detected in a southerly mainland area of NYS, in the Town of Thompson, Sullivan County.

## Discussion

### Surveillance

In this arbovirus surveillance program, the number of individual mosquitoes and number of pools submitted annually remained consistent (Table 4, Fig. 1).

Among 57 counties in NYS (excluding NYC), between nine and 43, inclusive, participated in annual mosquito submission. The introduction of WNV to NYS in 1999 [31] resulted in more counties (range 13-43, average 25) opting to conduct mosquito and arbovirus surveillance, beginning in 2000 [17]. Testing mosquitoes from a greater number of counties did not result in more detections of EEEV, as detections of EEEV occurred in only six counties.

### Species of mosquitoes with virus

In NYS, this was the first time that the mosquito species *Cx. salinarius*, *Ps. ferox*, and *An. punctipennis* were found to have EEEV (Table 3).

The preponderance of detections of EEEV being in *Cs. melanura* is expected due to the importance of ornithophilic mosquito species such as *Culiseta* to maintain EEEV in a mosquito-wild bird cycle and the ecological setting where EEEV is present. Given the large percentage of *Cs. melanura* having EEEV, it is to be expected that the greater the number of *Cs. melanura* collected, the greater the number of pools having EEEV in years when EEEV is present (Fig. 2).

The preponderance of detections of EEEV being from mosquitoes collected in Madison, Oneida, Onondaga and Oswego Counties is consistent with the history of EEEV detections, observed from 1971 to 1992 [7, 11, 32].

In aggregate, from 1971 to 2012, in NYS there were 629 detections of EEEV in 11 mosquito species within seven genera. In order of frequency, 547 were from *Cs. melanura*, 27 from *Cq. perturbans*, 21 from *Cs. morsitans*, 14 from *Ae. canadensis*, seven from *Cx. pipiens-restuans* group, five from *Ae. vexans*, three from *An. quadrimaculatus*, and one each from *Cx. pipiens*, *Cx. restuans, Cx. salinarius*, *Ps. ferox* and *An. punctipennis*.

### Geographical distribution of mosquitoes with virus

During the period of 1971 to 1992, inclusive, four contiguous counties (Madison, Oneida, Onondaga and Oswego), have had more detections of EEEV in mosquitoes than any other location in NYS [7, 16]. This trend continued from 2003 to 2012.

In Suffolk County, EEEV was first detected in mosquitoes in 1973 [14]. Suffolk County is the easternmost county in NYS that EEEV was detected (Town of East Hampton), in 1996 and 2003. The neighboring state of Rhode Island reported one case of human EEE, on Block Island (in Washington County) in 1993 and 71 detections of EEEV in 1996, 1997, 1998 and 2000 in *Cs. melanura*, *Cq. perturbans*, *Ae. canadensis* and *Ae. vexans* and unidentified *Anopheles* species and *Culex* species in Washington County and Newport County [33, 34]. These detections in Rhode Island are comparable to our detections in Suffolk County during that time (Table 3). The coast of Block Island is 22 km off the northeast coast of Suffolk County. The neighboring state of Connecticut reported EEEV in eight mosquito species in New London County in 1996 [35], again comparable to our detections in Suffolk County in the period 1993 to 1997 (Table 3). The coast of New London County is 13 km off the northern coast of Suffolk County.

In Sullivan County, EEEV was first detected in mosquitoes in 2004, before which time EEEV had not been reported in mosquitoes or vertebrates. In the Town of Thompson in Sullivan County, EEEV was detected in *Cs. melanura* (Table 3), collected on September 2, 2004. Sullivan County had its first case in an eagle on September 7, 2004 and its first case in a horse on October 3, 2004 [36]. One of these cases was located 1 km from where the EEEV-infected *Cs. melanura* were collected. In adjacent Ulster County, there was a case in a horse on September 15, 2004 and a case in a goldfinch on October 19, 2004 [36].

### Timing of mosquitoes with virus

In NYS, detection of EEEV in mosquitoes every year from 2003 to 2012, inclusive, was the longest consecutive time period, to date. During the 20-year period from 1993 to 2012, there was a five-year period from 1998 to 2002 without detection of EEEV in mosquitoes.

In Madison, Oneida, Onondaga and Oswego Counties, during the 22-year period from 1971 to 1992, there was a three-year interval, from 1984 to 1986, without detection of EEEV in mosquitoes [7, 16]. From 1993 to 2012, EEE in humans was reported in only Onondaga or Oswego counties, in 2009, 2010 and 2011 [36].

In 2003 concomitantly, in Suffolk County, there were EEEV-infected mosquitoes and an EEE case in a horse [36]. In other years when there were detections of EEEV in mosquitoes, specifically 1993, 1994, 1996, 1997 and 2008, there were no cases of EEE in horses or other vertebrates reported in Suffolk County [36].

In Massachusetts, EEEV was detected in mosquitoes every year from 1968 to 1980 and 1982 to 1993 [37, 38] and every year from 1997 to 2001 [38]. In New Jersey, EEEV was detected in mosquitoes every year from 2003 to 2010 [39]. In Connecticut, EEEV was detected in mosquitoes every year from 2009 to 2013 [40].

The calendar date of the earliest seasonal detection of EEEV in mosquitoes was June 25, in 1991, during the *previous* 22-year surveillance period of 1971 to 1992 [7, 16]. The calendar date of the earliest seasonal detection of EEEV in mosquitoes was also June 25, in 2007, during the *present* 20-year surveillance period of 1993 to 2012.

The finding of only two detections in October (Table 5) is consistent with the natural seasonal decrease in active adult mosquitoes in NYS, the typical onset of mosquito-killing frosts and the conclusion of seasonal mosquito surveillance programs.

### Transmission of EEEV from mosquitoes

Theoretically, any of the 11 mosquito species testing positive for EEEV may play a role in transmission to mammals. Hayes & Doane [41] proposed a role for *Cs. melanura* in transmission to mammals. *Culiseta melanura* has a higher percentage of specimens carrying EEEV than any other mosquito species [42], which has been used to argue for the involvement of this species in enzootic [1] and epidemic [16] transmission of EEEV. The identification of human blood in field-caught *Cs. melanura* has been used to argue for the involvement of this species in transmission of EEEV to humans [4, 5]. *Aedes albopictus* (Skuse), *Ae. vexans*, *Ae. canadensis* and *Cq. perturbans*, have been shown to carry EEEV [8].

*Culex salinarius*, *Ps. ferox* and *An. punctipennis* may feed on humans [24]. *Culex salinarius*, *Ps. ferox* and *An. punctipennis* can seek hosts during the usual season of EEE disease in NYS, which has been July to October [7, 32, 36]. EEEV was detected in *Cx. salinarius* and *Ps. ferox* in Mississippi in 1998, 1999 and 2002 [43]. EEEV was detected in *An. punctipennis* in South Carolina between 1996 and 1998 [44]. EEEV was detected in *Cx. salinarius* in Ohio state in 1991 concomitantly with cases of EEE in horses [45]. *Culex salinarius* and *An. punctipennis* are considered likely vectors of EEE in Massachusetts [46]. Vaidyanathan et al. [47] found *Cx. salinarius* and *An. punctipennis* to be susceptible to infection with EEEV *via* mouth parts and found these species to have potential for salivary transmission. *Psorophora ferox* is a known vector of Venezuelan equine encephalitis virus (VEEV) [48], which suggests *Ps. ferox* has the potential to transmit EEEV.

### Practical applications

During this surveillance, every year, in the usual course of official duty, the state health department presented information on the presence of mosquitoes with EEEV to the appropriate officials in the affected counties. The officials, pursuant to public health code, made decisions on when and how to prevent disease by implementing a public health information campaign *via* newspapers, radio, television or web sites. Also, officials decided when and how to implement vector control using mosquito adulticides in affected geographical areas. The state health department presented this information on the presence of mosquitoes with EEEV to practicing veterinarians to consider prevention, such as vaccination and to consider the diagnosis of EEE.

### Limitations of this study

This study was not able to determine the latest date in the year that EEEV was present in mosquitoes, because the county and state mosquito surveillance programs ceased in October. Each county health department made its own decision to participate, or not, in surveillance. The number of participating counties varied from year to year. Counties could not submit all collected mosquitoes, due to testing capacity. The collected mosquitoes that were not submitted may have contained EEEV. Choices of trap types by each county may be viewed as sampling bias, because different traps attract different species to different extents. EEE disease in vertebrate animals, including humans, was reported in 15 counties during 2003 to 2012 [36]. Of these 15 counties, 11 to 12 counties did routine annual adult mosquito surveillance; two counties did no surveillance.

During years 2000 to 2008, when the emergence of WNV was a concern, more counties participated (Table 1). Nevertheless, each year, similar numbers of mosquitoes were submitted, and similar numbers of pools were made and tested for the presence of EEEV in mosquitoes (Table 4 and Fig. 1).

Vero cell culture testing may fail to detect virus, if the specimen is degraded, or the virus is not viable. RT-PCR testing may fail to detect virus, if the specimen is degraded, or there is an unrecognized mutation in the virus. In 2000, RT-PCR was established for surveillance of WNV in mosquitoes following the introduction to NYS of WNV disease. This allowed for EEV to be tested by RT-PCR, along with WNV, in a single multiplex. From 1993 to 2012, all pools of all mosquitoes, except *Cx. pipiens* and *Cx. restuans*, were tested on Vero cell culture. If a pool of *Cx. pipiens* and *Cx. restuans* tested positive by RT-PCR, it was then tested by Vero cell culture. In all years, our specimens were tested on Vero cell culture, the sensitivity of which was not expected to change. The consistent use, throughout this surveillance, of a standard culture method means the presence of EEEV in any year is not negated by the addition or modification of a RT-PCR assay.

## Conclusions

This was the first time in NYS that EEEV was detected in *Cx. salinarius*, *Ps. ferox* and *An. punctipennis*. The detection of EEEV in mosquitoes every year for 10 years was the longest time span since surveillance began in 1971. This was preceded by a period of five years of no EEEV detections. The calendar date of the earliest annual appearance of EEEV in mosquitoes did not change during surveillance spanning 42 years.

## References

[1] Howard JJ, Wallis RC (1974). Infection and transmission of Eastern equine encephalomyelitis virus with colonized *Culiseta melanura* (Coquillett). Am J Trop Med Hyg..

[2] Nasci RS, Edman JD (1981). Blood-feeding patterns of *Culiseta melanura* (Diptera: Culicidae) and associated sylvan mosquitoes in southeastern Massachusetts Eastern equine encephalitis enzootic foci. J Med Entomol..

[3] Molaei G, Oliver J, Andreadis TG, Armstrong PM, Howard JJ (2006). Molecular identification of blood-meal sources in *Culiseta melanura* and *Culiseta morsitans* from an endemic focus of Eastern equine encephalitis virus in New York. Am J Trop Med Hyg..

[4] Apperson CS, Hassan HK, Harrison BA, Savage HM, Aspen SE, Farajollahi A (2004). Host feeding patterns of established and potential mosquito vectors of West Nile virus in the eastern United States. Vector Borne Zoonotic Dis..

[5] Molaei G, Andreadis TG, Armstrong PM, Thomas MC, Deschamps T, Cuebas-Incle E (2013). Vector-host interactions and epizootiology of Eastern equine encephalitis virus in Massachusetts. Vector Borne Zoonotic Dis..

[6] Hayes RO, Beadle LD, Hess AD, Sussman O, Bonese MJ (1962). Entomological aspects of the 1959 outbreak of Eastern encephalitis in New Jersey. Am J Trop Med Hyg..

[7] Howard JJ, Grayson MA, White DJ, Morris CD (1994). Eastern equine encephalitis in New York State. J Florida Mosq Control Assoc..

[8] Nasci RS, Mitchell CJ (1996). Arbovirus titer variation in field-collected mosquitoes. J Am Mosq Control Assoc..

[9] Armstrong PM, Andreadis TG (2010). Eastern equine encephalitis virus in mosquitoes and their role as bridge vectors. Emerg Infect Dis..

[10] Chamberlain RW, Sikes RK, Nelson DB, Sudia WD (1954). Studies on the North American arthropod-borne encephalitides VI. Quantitative determinations of virus-vector relationships. Am J Trop Med Hyg..

[11] Morris CD, Whitney E, Bast TF, Deibel R (1973). An outbreak of Eastern equine encephalomyelitis in upstate New York during 1971. Am J Trop Med Hyg..

[12] Morris CD, Caines AR, Woodall JP, Bast TF (1975). Eastern equine encephalomyelitis in upstate New York, 1972–1974. Am J Trop Med Hyg..

[13] Srihongse S, Grayson MA, Morris CD, Deibel R, Duncan CS (1978). Eastern equine encephalomyelitis in upstate New York: studies of a 1976 epizootic by a modified serologic technique, hemagglutination reduction, for rapid detection of virus infections. Am J Trop Med Hyg..

[14] Zaki MH (1979). Arthropod-borne viral encephalitides: Illusion or reality in Suffolk County?. N Y State J Med..

[15] Rochlin I, Harding K, Ginsberg HS, Campbell SR (2008). Comparative analysis of distribution and abundance of West Nile and eastern equine encephalomyelitis virus vectors in Suffolk County, New York, using human population density and land use/cover data. J Med Entomol..

[16] Howard JJ, Grayson MA, White DJ, Oliver J (1996). Evidence for multiple foci of Eastern equine encephalitis virus (Togaviridae: *Alphavirus*) in central New York State. J Med Entomol..

[17] Lukacik GL, Anand M, Shusas EJ, Howard JJ, Oliver J, Chen H (2006). West Nile virus surveillance in mosquitoes in New York State, 2000–2004. J Am Mosq Control Assoc..

[18] Newhouse VR, Chamberlain RW, Johnson JG, Sudia WD (1966). Use of dry ice to increase mosquito catches of CDC miniature light trap. Mosq News..

[19] Reiter P (1983). A portable battery-powered trap for collecting gravid *Culex* mosquitoes. Mosq News..

[20] Morris CD (1981). A structural and operational analysis of diurnal resting shelters for mosquitoes (Diptera: Culicidae). J Med Entomol..

[21] Moore CG, McLean RG, Mitchell CJ, Nasci RJ, Tsai TF, Calisher CH, et al. Guidelines for arbovirus surveillance programs in the United States. Fort Collins, CO, USA: United States Department of Health and Human Services. Centers for Disease Control and Prevention. 1993;

[22] White DJ, Kramer LD, Backenson PB, Lukacik G, Johnson G, Oliver J (2001). Mosquito surveillance and polymerase chain reaction detection of West Nile Virus, New York State. Emerg Infect Dis..

[23] Means RG. Mosquitoes of New York. Part I. The genus *Aedes* Meigen with identification keys to genera of Culicidae. Albany: New York State Museum (Bulletin 430a). 1979:1–221.

[24] Means RG (1987). Mosquitoes of New York. Part II. Genera of Culicidae other than *Aedes* occurring in New York. Albany: New York State Museum (Bulletin 430b).

[25] Stojanovich CJIllustrated key to common mosquitoes of northeastern North America. Atlanta: Published by Author; 1961. p. 1–49.

[26] Andreadis TG, Thomas MC, Shepard JJ. Identification guide to the mosquitoes of Connecticut. New Haven: Connecticut Agricultural Experiment Station (Bulletin 966). 2005:1–173.

[27] Boromisa RD, Grayson MA (1990). Incrimination of *Aedes provocans* as a vector of Jamestown Canyon virus in an enzootic focus of northeastern New York. J Am Mosq Control Assoc..

[28] Kauffman EB, Jones SA, Dupuis AP, Ngo KA, Bernard KA, Kramer LD (2003). Virus detection protocols for West Nile Virus in vertebrate and mosquito specimens. J Clin Microbiol..

[29] Lambert AJ, Martin DA, Lanciotti RS (2003). Detection of North American Eastern and Western equine encephalitis viruses by nucleic acid amplification assays. J Clin Microbiol..

[30] Hull R, Nattanmai S, Kramer LD, Bernard KA, Tavakoli NP (2008). A duplex real-time reverse transcriptase polymerase chain reaction assay for the detection of St. Louis encephalitis and Eastern equine encephalitis viruses. Diagn Microbiol Infect Dis..

[31] Lanciotti RS, Roehrig JT, Deubel V, Smith J, Parker M, Steele K (1999). Origin of the West Nile Virus responsible for an outbreak of encephalitis in the northeastern United States. Science.

[32] Howard JJ, Morris CD, Emord DE, Grayson MA (1988). Epizootiology of Eastern equine encephalitis virus in upstate New York, USA. VII. Virus surveillance 1978–85, description of 1983 outbreak, and series conclusions. J Med Entomol..

[33] Takeda T, Whitehouse CA, Brewer M, Gettman AD, Mather TN (2003). Arbovirus surveillance in Rhode Island: assessing potential ecologic and climatic correlates. J Am Mosq Control Assoc..

[34] Markowski D. Eastern equine encephalitis outbreak 1996: what really happened in Rhode Island. In, Proceedings of the 42nd Annual Meeting of the Northeastern Mosquito Control Association, 1996. 9–11 December 1996. Mystic, Connecticut. http://www.nmca.org/paper6a.htm. Accessed 25 Mar 2018.

[35] Andreadis TG, Anderson JF, Tirrell-Peck SJ (1998). Multiple isolations of Eastern equine encephalitis and highlands J viruses from mosquitoes (Diptera: Culicidae) during a 1996 epizootic in southeastern Connecticut. J Med Entomol..

[36] Oliver J, Lukacik G, Kramer LD, Backenson PB, Sherwood JA, Howard JJ (2016). Geography and timing of cases of Eastern equine encephalitis in New York State from 1992 to 2012. Vector Borne Zoonotic Dis..

[37] Edman JD, Timperi R, Werner B (1993). Epidemiology of Eastern equine encephalitis in Massachusetts. J Florida Mosq Control Assoc..

[38] Hachiya M, Osbourne M, Stinson C, Werner BG (2007). Human Eastern equine encephalitis in Massachusetts: predictive indicators from mosquitoes collected at 10 long-term trap sites, 1979–2004. Am J Trop Med Hyg..

[39] New Jersey Agricultural Experiment Station. Vector Surveillance Reports, 1977 to 2017. Center for Vector Biology, School of Environmental & Biological Sciences, Rutgers, The State University of New Jersey, New Brunswick, New Jersey. http://vectorbio.rutgers.edu/reports/vector/. Accessed 25 Mar 2018.

[40] Connecticut Agricultural Experiment Station. State of Connecticut Mosquito Trapping and Arbovirus Testing Program--Historical Information, 1998 to 2016. New Haven, Connecticut. http://www.ct.gov/caes/cwp/view.asp?a=2819&q=552822. Accessed 25 Mar 2018.

[41] Hayes RO, Doane OW (1958). Primary record of *Culiseta melanura* biting man in nature. Mosq News..

[42] Wallis RC (1971). Recent advances in research on the Eastern encephalitis virus. Yale J Biol Med..

[43] Cupp EW, Tennessen KJ, Oldland WK, Hassan HK, Hill GE, Katholi CR (2004). Mosquito and arbovirus activity during 1997–2002 in a wetland in northeastern Mississippi. J Med Entomol..

[44] Wozniak A, Dowda HE, Tolson MW, Karabatsos N, Vaughan DR, Turner PE (2001). Arbovirus surveillance in South Carolina, 1996–98. J Am Mosq Control Assoc..

[45] Nasci RS, Berry RL, Restifo RA, Parsons MA, Smith GC, Martin DA (1993). Eastern equine encephalitis virus in Ohio during 1991. J Med Entomol..

[46] Moncayo AC, Edman JD (1999). Toward the incrimination of epidemic vectors of eastern equine encephalomyelitis virus in Massachusetts: abundance of mosquito populations at epidemic foci. J Am Mosq Control Assoc..

[47] Vaidyanathan R, Edman JD, Cooper LA, Scott TW (1997). Vector competence of mosquitoes (Diptera: Culicidae) from Massachusetts for a sympatric isolate of Eastern equine encephalomyelitis virus. J Med Entomol..

[48] Scherer WF, Dickerman RW, Diaz-Najera A, Ward BA, Miller MH, Schaffer PA (1971). Ecologic studies of Venezuelan encephalitis virus in southeastern México III. Infection of mosquitoes. Am J Trop Med Hyg..

